# A new method of kidney biopsy using low dose CT-guidance with coaxial trocar and bard biopsy gun

**DOI:** 10.1186/1480-9222-15-1

**Published:** 2013-01-07

**Authors:** Xiao-ling Pi, Zhen Tang, Li-qian Fu, Mei-hua Guo, Mei-hua Shi, Lan Chen, Zheng-ying Wan

**Affiliations:** 1 Department of Nephrology, Pudong New Area Gongli Hospital, Shanghai, 200135, China; 2 Department of Radiology, Fengxian Center Hospital-Branch of Shanghai Sixth People’s Hospital, Jiaotong University, Shanghai, 201400, China

**Keywords:** Kidney biopsy, Low dose CT scanning, Bard biopsy gun, Coaxial trocar

## Abstract

**Background:**

To explore a new method of kidney biopsy with coaxial trocar and bard biopsy gun under low dose computed tomography (CT)-guidance and evaluate its accuracy, safety, and efficacy.

**Methods:**

Sixty patients underwent renal biopsy under CT-guidance. They were randomly divided into two groups: group I, low dose CT-guided (120 kV and 25 or 50 mAs) and group II, standard dose CT-guided (120 kV and 250 mAs). For group I, the coaxial trocar was accurately placed adjacent to the renal capsule of the lower pole, the needle core was removed, and samples were obtained with a bard biopsy gun. For group II, the coaxial trocar was not used. Total number of passes, mean biopsy diameter, mean glomeruli per specimen, mean operation time, mean scanning time, and mean radiation dose were noted. Dose-length product (DLP) was used to calculate the radiation doses. After 24 hours of the biopsy, ultrasound was repeated to identify any subcapsular hematoma.

**Results:**

Success rate of biopsy in group I was 100% while using low dose CT-guidance along with coaxial trocar renal. There was no statistic differences bewteen group I and II in the total number of passes, mean biopsy diameter, mean glomeruli per specimen and mean time of operation and CT scanning. The average DLP of group I was lower as compared to the value of group II (*p* <0.05).

**Conclusions:**

Kidney biopsy using coaxial trocar and bard biopsy gun under low dose CT was an accurate, simple and safe method for diagnosis and treatment of kidney diseases. It can be used for repeat and multiple biopsies, particularly suitable for obese and renal atrophy patients in whom the kidneys are difficult to image.

## Background

Kidney diseases have been a silent killer with rising incidence worldwide and poor outcomes [1]. Early diagnosis and treatment can prevent the complications of decreased kidney function and reduce the risk of concomitant cardiovascular disease [2,3]. However, many of the kidney diseases are undiagnosed by non-invasive methods and renal pathological examination has indicated to establish definitive histopathological diagnosis in diffuse renal disease [4].

Renal biopsy is an immensely valuable tool to determine the cause of the disease, predict the prognosis, and direct treatment which includes open biopsy (laparoscopically [5], transvenous [6] and percutaneous biopsy Ultrasonograpgy [7], CT [7,8], or magnetic resonance imaging (MRI) [9]. The only disadvantage with these methods could be the severe surgical trauma [10]. Ultrasound-guided percutaneous renal biopsy has traditionally been used to obtain biopsies but the maintenance of sterilization is very difficult with the use of coupling agents [11-13]. In patients with chronic renal failure and renal atrophy caused by glomerulosclerosis and interstitial fibrosis, the interface of renal cortex and medullary is unclear under ultrasound images [14]. Similarly, biopsy procedures could be unsuccessful in obese patients where imaging of the kidneys is difficult [15]. Thus, the puncture operation would become inconvenient leading to an inaccurate positioning. The diameter of biopsy tissues is proportional to the number of glomeruli in the renal cortex hence, renal cortex becomes an adequate site for the biopsies [16]. This makes accurate positioning essential. Conventional CT-guided renal biopsy is extremely accurate but high radiation poses a major problem. Hence, CT-guided renal biopsy has been reserved for more difficult cases [4]. However, significant changes in the biopsy design as well as use the minimum radiation dose to achieve a reasonable diagnostic image quality may further enhance the accuracy, safety, and efficacy of CT-guided kidney biopsy. To understand the scope of CT-guided biopsy techniques the present study was conducted to investigate the utility of a low radiation, minimally invasive, and accurate biopsy method using a low dose CT-guided coaxial trocar and automatic biopsy gun.

## Results and discussion

The cores of 13.5 mm (range 9–31 mm) from cortico-medullary junction were collected with a biopsy needle in compliance with the diagnostic requirements. Each core contained mean glomeruli per specimen 15.3 (range 12–37) and 15.2 in the group-I and II, respectively. The mean operation and scanning time was 13 minutes (range 10–13 minutes) and 4.1 sec (range 3.935-5.268 sec), respectively in the group I. The mean operation and scanning time was 13.5 minutes and 4.2 sec, respectively in the group II. There was no significant difference between the two groups with the five main parameters (*p* >0.05) (Table 1). The total average DLP of group I (311.5 mGy × cm) was low as compared to that of group II (1166.3 mGy × cm) and difference was statistically significant (*p* <0.05). The total average DLP of group I was less than 1/3 of the group II DLP. The decrease in the total average DLP for group I was achieved by modifying the tube current-time product (mAs).

**Table 1:** Comparison of low-dose CT-guided and standard-dose CT-guided renal biopsy

	**Total number of passes**	**Mean biopsies diameter(mm)**	**Mean glomeruli per specimen**	**Mean operation time (M)**	**Mean scanning time (S)**	**Mean radiation dose (mGy.cm)**
low-dose group (30cases)	62	13.5 ± 0.8	15.3 ± 1.0	13.2 ± 2.0	4.1 ± 1.0	311.5 ± 9.8
Standard dose group(30cases)	64	13.8 ± 0.7	15.2 ± 1.1	13.5 ± 2.1	4.2 ± 1.1	1166.3 ± 10.4
P value, student’s *t*-test	>0.05	>0.05	>0.05	>0.05	>0.05	<0.01

## Complications

Routine urine for three consecutive times showed that all of the patients experienced microhematuria after biopsy, there were no patients with gross hematuria. Two patients in the group I (6.7%) and three patients in the group II (10%) had small sub capsular hematoma 24 hours after biopsy, but all of them remained asymptomatic, without any medical treatment.

## Discussions

Kidney biopsy under CT-guidance has been the gold standard for diagnosis and treatment of glomerular and tubulo-interstitial disease [17,18]. Computed tomography provides detailed view of anatomical structures, which helps localization of even small lesions located in the kidneys. It helps in accurate planning of the needle biopsy, avoiding inadvertent puncture of vascular structures [19]. Computed tomography guided kidney biopsies can shorten the period of hospital stay and decrease the number of operations as well as the treatment costs [20]. As there is a risk of high dose radiation exposed complications, as well as multiple or repeated examinations are very likely required for patients suffered of chronic kidney failures, methods for its protection have drawn increasing attention from researchers and clinicians. Although alternative imaging technologies such as ultrasound do not pose risk of radiation, the difficulties of maintaining sterilization with coupling agents and having clear views with ultrasound imaging in patients with chronic renal failure caused by glomerulosclerosis and interstitial fibrosis restrict their usages in renal biopsy. In CT scanning, the minimum radiation dose should be as low as reasonably achievable for maintenance of diagnostic quality [21,22]. In our study, we reduced the radiation dose by decreasing tube current-time from 250 mAs to 80mAs, which was less than 1/3 of the standard dose of CT. The average DLP for group I was 311.5 mGy × cm, which was only 26.7% of the standard dose of CT and was almost negligible. Moreover, use of this method could result in good diagnostic image quality for routine clinical diagnostic applications.

Generally, CT-guided coaxial trocar renal biopsy is easy to operate, ensures the sterility of entire visual field, and helps in reduction of the risk of infection. The number of glomeruli in the cortex is critical for the diagnosis of kidney disease; generally a minimum of 5 to 10 intact glomeruli would satisfy the requirements [4,18]. Biopsy tissues diameter is proportional to the number of glomeruli in the renal cortex so the biopsies must be taken from renal cortex. Therefore, an accurate positioning under CT-guidance is required. In our study, low dose CT scan is a bit weak, but it can clearly distinguish the kidney cortex and medulla. The mean glomeruli per specimen were 15.3, which could be successfully analyzed by light microscopy, immunofluorescence, and electron microscopy. The operation time was less (10 to 15 minutes per patient; the median time was 13 min) compared with earlier reported studies [14,18]. Some of these studies reported that in order to reduce hemorrhage complications, 18-gauge biopsy needle and 3 cores were generally required for pathologic diagnosis [14]. In our study, adequate tissue was obtained using the 16-gauge biopsy needle and only 2 cores were needed. The pathological diagnosis rate was 100%, which was slightly higher than related previous studies [4,18]. The biggest advantage of coaxial trocar renal biopsy under thin-layer scan was the accurate positioning, safe operation, and high successful rate. The majority of the studies reported the use of 10 mm axial CT scans [4]. In our study 5 mm axial CT scans were used to provide more accurate positions. The location of puncture needle could be determined by coaxial trocar before biopsy, which is similar to having renal biopsy under direct vision. Thus, multiple biopsies could be possible with only one positioning. This is convenient, efficient, and helps in reducing the radiation exposure.

The biopsy gun provided high-quality specimen with little bleeding and rapid action. The action of biopsy gun may be slowed down if used alone, due to the resistance of lumbar muscles in patients. This could result in low-quality specimen and increased chances of bleeding [4]. Nonetheless, in coaxial trocar assisted biopsy, the bard biopsy gun passes through a trocar to minimize the frictional resistance between the biopsy needle and the trocar.

In conclusion, low dose CT-guided renal biopsy was accurate, safe, and effective. It had the advantages of clearly displaying the regional anatomic location and structure successfully with no or few complications. While this technology is beneficial to all sorts of patients with chronic renal failure or renal atrophy as the interface of renal cortex and medulla is much clear in low-dosed CT scanning, it is especially useful to obtain enhanced images in patients who were obese or had renal atrophy.

## Methods

### Patients and medical history

We studied 60 patients, who underwent renal biopsies at the Department of Nephrology, Pudong New Area Gongli Hospital (Shanghai, China) from October 2009 to December 2010. Prior to each procedure, the risks and benefits of these biopsies were discussed, and informed consent was obtained from each patient. This study was approved by ethics committees of Shanghai Pudong New Area Gongli Hospital and Fengxian Center Hospital-Branch of Shanghai Sixth People’s Hospital. In this study 29 male and 31 female patients were included. The median age of patients were 51 years (range 23–80 years) with a medical history of nephritic syndrome (28 patients, 46.66%), acute renal failure (5 patients, 8.33%), hypersensitivity nephropathy (4 patients, 6.66%), microscopic hematuria (2 patients, 3.33%), interstitial nephritis (2 patients, 3.33%) and six patients each of diabetic nephropathy, chronic renal failure with obesity, and proteinurea. Out of 28 nephrotic syndrome patients, one was with massive ascites and two were obese. From six chronic renal failure patients, three had kidney length diameter less than 9 cm.

### Inclusion criteria

Inclusion criteria was urine protein (+) or 24 hour urinary protein excretion more than 1.0 grams; unexplained acute renal failure (serum creatinine: 133 μmol~500 μmol); acute exacerbation of chronic renal failure (serum creatinine: 133 μmol~500 μmol); kidney diameter ≥8 cm; and glomerular hematuria (gross hematuria or severe microscopic hematuria). Patients who did not meet the above criteria were excluded. Before biopsy, all coagulation indicators were within the normal range and blood pressure levels of patients were maintained below 140/90 mmHg. During biopsy, local anesthetic delivery was similar to the one for conventional puncture. Also, CT scan images of kidney were obtained to figure out the number, size, morphology, and measure the depth from the skin to the dorsal renal capsule.

### Randomization

All The patients were randomized evenly to group I-to receive low dose radiation (120 kV and 25 or 50 mAs) and group II-to receive standard dose radiation (120 kV, 250 mAs) (30 patients in each group). We used dose-length product (DLP) as a dose descriptor indicator. It characterizes the total ionizing energy imparted to the reference phantom for a given examination and can be used to evaluate the radiation doses received by the patients. All biopsies were performed under CT (Philips Brilliance 16 CT scanner) guidance using bard biopsy gun (16-gauge × 16 cm), self-made coaxial trocar (20-gauge trocar, 16-gauge needle core), self-made U-shaped locator, and 7-gauge needle (6 cm). The renal biopsies were performed by a full-time nephrology faculty experienced in the technique.

**Figure 1:**
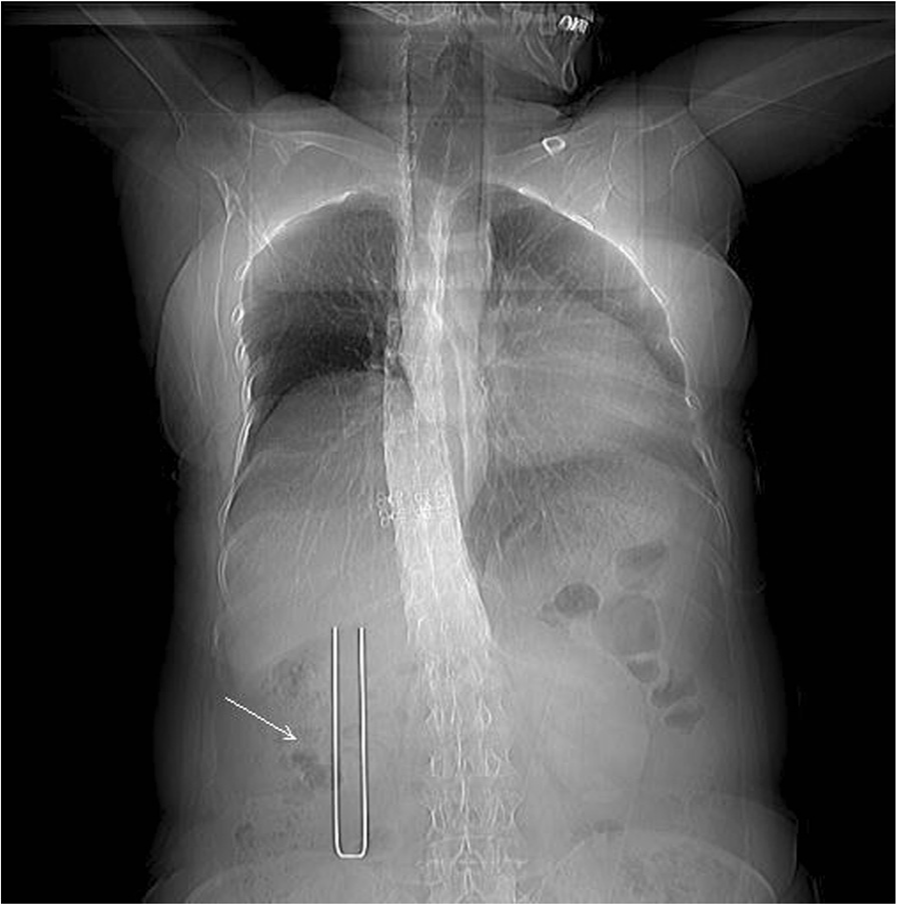
The U-shaped locator (arrow) was placed for skin positioning (topography).

**Figure 2:**
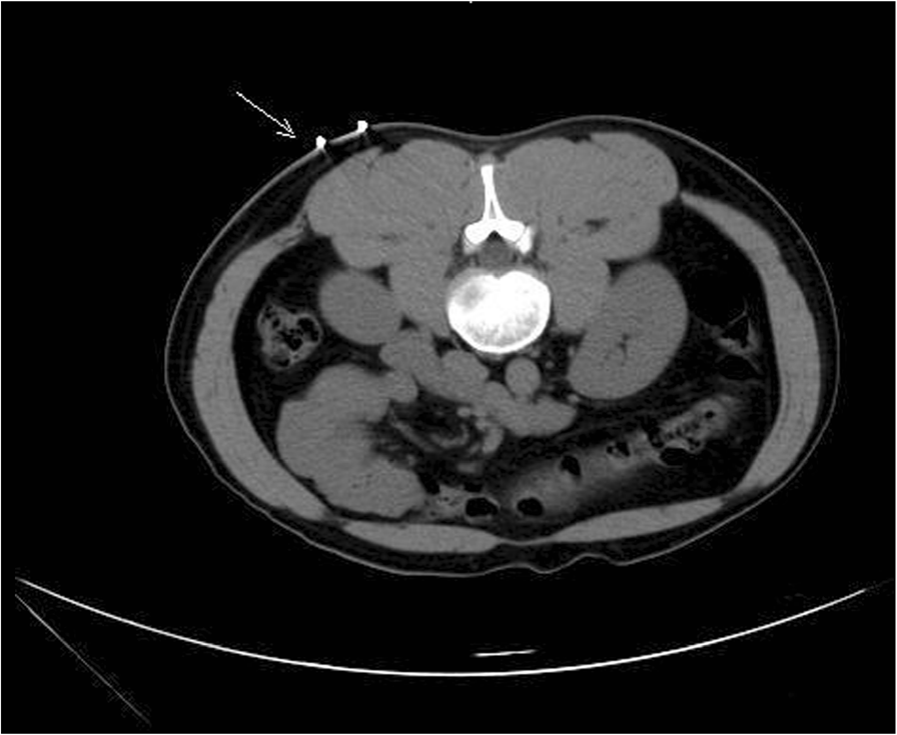
5.0 mm axial CT scan was taken through the lower pole of the kidney to determine the puncture site (Standard-dose scanning).

**Figure 3:**
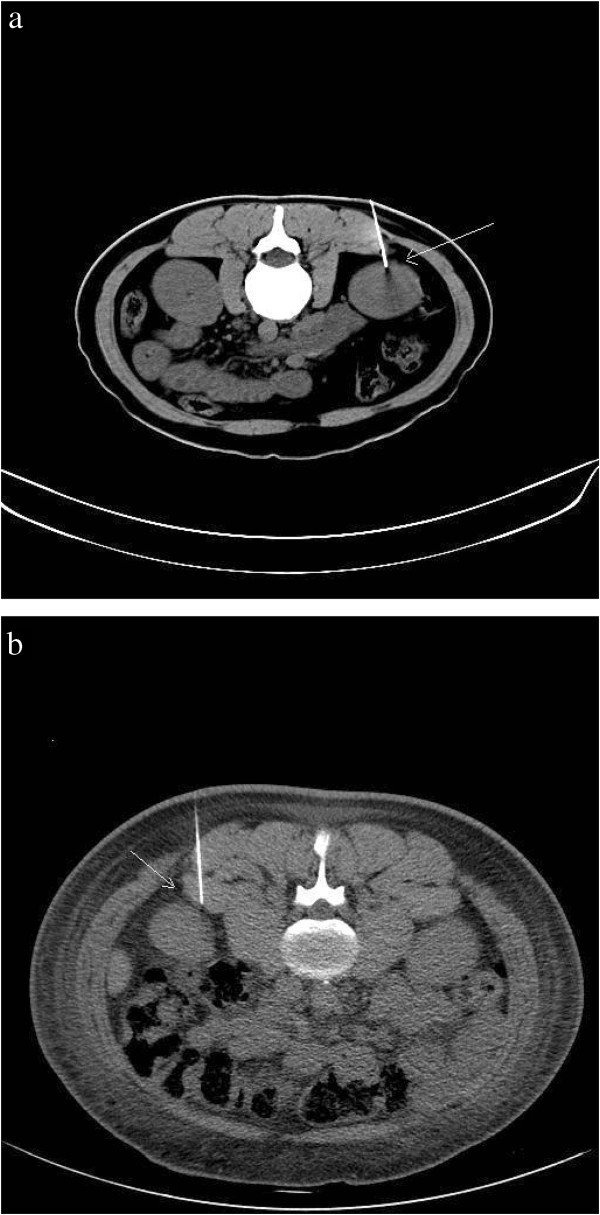
**a: A long needle (arrow) was used to deliver local anesthesia to the subcutaneous and perirenal tissues down to the renal capsule (Standard-dose scanning).b**: a long needle (arrow) was used to deliver local anesthesia to the subcutaneous and perirenal tissues down to the renal capsule (Low-dose scanning).

**Figure 4:**
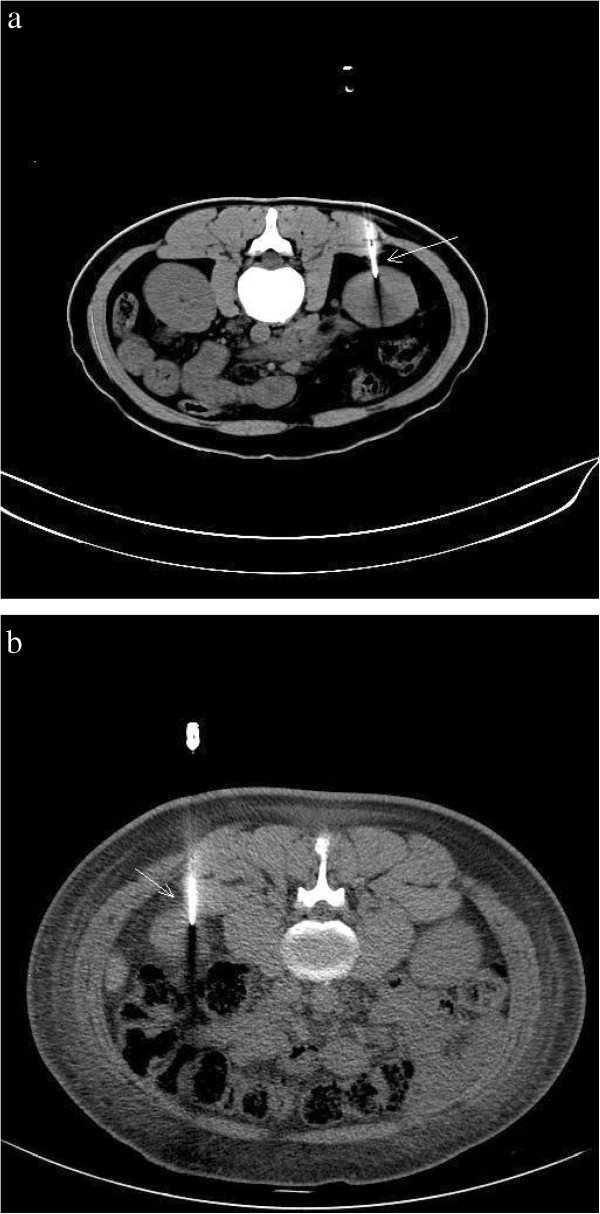
**a: The coaxial trocar (arrow) was placed to the renal capsule, ready for renal biopsy.** (Standard-dose scanning) **b**: The coaxial trocar (arrow) was placed to the renal capsule, ready for renal biopsy. (Low-dose scanning).

After defecation/urination, the patient was instructed to lie down in the appropriate position on the CT scanner table and a soft pillow was placed under the abdomen as support. The patient was instructed not to move during the procedure. Under CT-guidance a U-shaped locator was placed at the puncture site of the lower pole and approximately 6–7 cm parallel to the spine (Figure 1). The puncture site was fully covered and patients were asked to hold their breath, after which a 5.0 mm axial CT scan was taken through the lower pole of the kidney (Figure 2). The laser scan line was used as an anchor tag. The cross point of the laser scan lines and the U-shaped locator was localized for biopsy and the puncture site was marked with a marker pen. The puncture site was sterilized with compound iodine solution, a sterile drape was placed over the site, and local anesthesia was administered to the subcutaneous and perirenal tissues down to the renal capsule using 7-gauge needle. The distance from the skin to the renal cortex was measured and a second CT scan was performed (Figure 3). Later the 7-gauge needle was pulled out and a small incision on the puncture site was made with a small sharp knife. The coaxial trocar was placed to the renal capsule in the same path of the anesthetic needle and rescanned (Figure 4). The style of the coaxial trocar was removed and replaced with an activated bard biopsy gun. The biopsy was then done immediately by triggering the biopsy gun during suspended respiration.

After biopsy, the biopsy gun was pulled out immediately; all the specimens inside the needle were removed and placed on ice-cold saline-soaked gauze for pathologic examination. When the specimens were not sufficient to establish the diagnosis, repeat biopsies were performed using the same technique as the first. The needle was removed after the tissue sample was taken; gauze was placed on the site for 3 minutes to stop the bleeding and cores were sent for pathological analysis. After the biopsy, patients were asked to stay in the bed for 24 hours. At 24 hours after the biopsy, ultrasound was repeated to identify any subcapsular hematoma. Total number of passes, mean biopsies diameter, mean glomeruli per specimen, mean operation time, mean scanning time, and mean radiation dose were recorded. Statistical significance between groups was assessed by student’s *t*-test and *p* <0.05 was considered to be threshold for statistical significance.

## Competing interests

The authors declare that they have no competing interests.

## Authors’ contributions

XLP conceived of the study and participated in operations of all patients. ZT was Guarantor of integrity of the entire study and participated in its design and Data acquisition. LQF and MHG screened patients and participated in operations of some patients. MHS, LC and ZYW participated preoperative and postoperative observation. All authors read and approved the final manuscript.
